# Y-Linked Expression Signatures Distinguish Dysfunctional Testicular States in Sheep

**DOI:** 10.3390/ani16132107

**Published:** 2026-07-07

**Authors:** Yangkai Liu, Yali Song, Jialei Chen, Wanhong Li, Xiangpeng Yue

**Affiliations:** State Key Laboratory of Herbage Improvement and Grassland Agro-Ecosystems, Key Laboratory of Grassland Livestock Industry Innovation, Ministry of Agriculture and Rural Affairs, College of Pastoral Agriculture Science and Technology, Lanzhou University, Lanzhou 730020, China; nwafuliuyangkai@126.com (Y.L.); songyl1015@163.com (Y.S.); chenjl2023@lzu.edu.cn (J.C.); limh@lzu.edu.cn (W.L.)

**Keywords:** sheep, Y chromosome, gametologs, testis development, cryptorchidism

## Abstract

The sheep Y chromosome encodes genes essential for testicular development and spermatogenesis, yet their transcriptional dynamics across developmental and pathological states remain largely uncharacterized. Here, we profiled Y-linked gene expression using RNA-seq datasets from Hu sheep testes across postnatal development (0, 3, 6, 12 months) and from divergent testicular (large, normal, small, cryptorchid) and epididymal (large, small) weights. Developmental trajectory analysis of 134 expressed Y-linked genes revealed three distinct patterns: progressive activation from birth to sexual maturity (dominated by multicopy families *ZNF280BY*, *HSFY*, *PRAMEY*, *TSPY3*), progressive decrease, and stage-specific expression. Differential expression identified 41 Y-linked genes consistently downregulated in small, cryptorchid, and pre-pubertal testes compared with normal or large mature testes. Hierarchical clustering showed that 6-month-old small testes transcriptionally clustered with cryptorchid testes, not with immature or normal testes. In the epididymis, only one Y-linked gene was differentially expressed between large and small groups. Among X–Y gametolog pairs, most showed positive expression correlations in testes, whereas *DDX3X/DDX3Y* exhibited a significant negative correlation (ρ = −0.450, *p* = 0.0185); strikingly, this pair reversed to a positive correlation in the epididymis (ρ = 0.607, *p* = 3.29 × 10^−5^). In conclusion, Y-linked transcriptional profiles distinguish testicular pathology from normal maturation, and *DDX3X/DDX3Y* regulation is strictly tissue-specific.

## Simple Summary

The Y chromosome contains genes that are crucial for male reproduction, but how these genes behave during testicular development is not well understood in sheep. In this study, we examined the expression of Y-chromosome genes in the testis-related tissues of Hu sheep at different ages and with different testis sizes, including cryptorchidism (testes that fail to descend). We found that several Y-linked genes, especially those present in multiple copies, become more highly expressed in the testes that mature normally. Interestingly, small testes in adult rams showed gene activity patterns similar to those of cryptorchid testes, rather than resembling immature or normal testes. This suggests that a reduced testis size in mature rams is not simply delayed development but may indicate a health problem. We also discovered that a specific pair of genes located on the X and Y chromosomes—called *DDX3X* and *DDX3Y*—behave differently in the testis compared with the epididymis, a nearby reproductive organ. These findings help us better understand how the sheep Y chromosome works and may provide us with new molecular tools to evaluate testicular health and male fertility in sheep.

## 1. Introduction

The mammalian male-specific region of the Y chromosome (MSY) is gene-rich, with genes essential for male sex determination, testicular differentiation, and spermatogenesis [1,2,3,4]. Unlike autosomes, it is characterized by extensive sequence repetition and multicopy gene families [5,6,7]. These multicopy Y-linked genes are predominantly expressed in the testis, reflecting their specialized roles in male reproduction [8,9]. In addition to multicopy genes, the Y chromosome also harbors evolutionarily conserved single-copy genes [10], including *SRY*, *EIF2S3Y*, *RBMY*, and *DDX3Y*, etc., that are critical regulators of male reproductive functions [4,11]. Many of these single-copy genes retain X-linked gametologs (X–Y pairs) originating from ancestral autosomes, and these pairs have increasingly diverged in expression patterns and shape sex-biased biology [12]. Similar associations between Y-linked variations and semen quality, sperm output, and reproductive performance have also been reported in livestock [13,14]. Despite these advances, the transcriptional organization and developmental dynamics of Y-linked genes in sheep testes remain poorly understood.

Meanwhile, publicly available sheep transcriptomic resources now encompass multiple reproductive contexts, including postnatal developmental stages, testicular size variation, cryptorchidism, and epididymal development [5,15], providing an unprecedented opportunity for cross-contextual comparative analysis. However, previous studies have largely focused on autosomal regulatory networks associated with testicular growth and spermatogenesis [16,17,18,19]. For example, hundreds of differentially expressed autosomal and X-linked genes of sheep testis have been identified as being involved in testis development [17], hormone regulation [20], and spermatogenesis pathways [17], while Y-linked transcriptional dynamics across reproductive states have received comparatively limited attention.

Postnatal testicular development involves extensive transcriptional remodeling accompanying germ-cell proliferation, meiotic initiation, and spermatogenic maturation [20,21]. These developmental transitions are tightly coupled with marked changes in testicular growth and reproductive capacity [20]. Studies in rams have demonstrated strong correlations among sperm output, epididymal sperm reserves, and scrotal circumference, indicating testis size is an important indicator of spermatogenic efficiency [22,23,24]. Conversely, pathological conditions such as cryptorchidism disrupt normal spermatogenesis and frequently result in severe reproductive impairment [25,26,27]. Although divergent testicular phenotypes have been extensively investigated at the histological and endocrine levels, their Y-linked transcriptional characteristics remain largely unexplored. In particular, whether sexually mature small testes resemble immature developmental stages or exhibit transcriptional characteristics distinct from the canonical maturation trajectory remains unclear.

In the present study, we integrated multiple sheep reproductive transcriptomic datasets to systematically characterize transcriptional landscapes of the sheep Y chromosome across developmental, morphological, and pathological testicular contexts. By focusing on multicopy and single-copy Y-linked genes, as well as X–Y gametolog pairs, we aimed to investigate developmental expression dynamics, reproductive-state-associated transcriptional variation, and potential transcriptional divergence associated with abnormal testicular phenotypes. This study provides a comprehensive framework for understanding Y-linked transcriptional regulation during sheep testicular development and reproductive dysfunction.

## 2. Materials and Methods

### 2.1. Data Collection and Phenotypic Grouping

A total of 78 Hu sheep samples were included in this study (Table S1). Among these, 23 RNA-seq datasets were retrieved from our lab’s previously published work [17,27], consisting of 11 testicular samples covering a developmental gradient (0-month, *n* = 3; 3-month, *n* = 3; 6-month, *n* = 3; 12-month, *n* = 2) and another 12 testicular samples from 6-month-old Hu sheep. The remaining 55 samples were newly collected from 6-month-old Hu sheep at the Experimental Station of Lanzhou University (Wuwei, China). This cohort included 40 epididymal tissues collected between August and October 2022 and 15 testicular tissues (collected between August and October 2021), which were categorized as follows: one case of cryptorchid testis, two normal-weight testes, six large-weight testes, and six small-weight testes. All samples were processed for RNA-seq library construction following our established protocols [27]. Notably, the testicular and epididymal tissues were collected from different individuals; thus, tissue-specific comparisons were conducted at the group level rather than as paired analyses. All historical samples and newly generated samples were raised under identical feeding formulas, housing environments and standardized tissue dissection procedures, which substantially reduce biological heterogeneity.

For group comparisons, only 6-month-old testicular and epididymal samples were adopted for statistical analysis based on total testicular weight: small testes (*n* = 6; ST < 90 g) and large testes (*n* = 6; LT > 410 g). In addition, normal testes (NT, *n* = 10) and cryptorchid testes (CT, *n* = 5) were classified based on anatomical position (normally descended vs. undescended testes). Epididymal groups were defined as large epididymides (*n* = 20; LE > 45 g) and small epididymides (*n* = 20; SE < 30 g). Detailed phenotypic parameters are provided in Table 1. Separately, the developmental gradient samples (0–12 months) were analyzed independently to assess puberty-related transcriptional shifts: samples aged 0–3 months were defined as pre-pubertal (PP), and those aged 6–12 months as sexually mature (SM) [23].

### 2.2. Histological Examination

To validate the weight-based phenotypic classification, representative testicular tissues from one ST individual (72.8 g) and one LT individual (467.4 g) were selected for hematoxylin and eosin (H&E) staining. Both individuals were from the LT and ST groups described in Section 2.1. Staining was performed following the protocol described in the previous study [28]. Briefly, samples were fixed in 4% paraformaldehyde, dehydrated through graded ethanol, cleared in xylene, and embedded in paraffin. Sections were cut at 5 μm thickness, mounted on slides, and stained with H&E. Stained sections were examined using an Olympus VS120 digital slide scanner (Olympus, Tokyo, Japan), and images were captured for morphological comparison.

### 2.3. RNA Extraction, Library Preparation, and RNA-Seq Data Processing

Total RNA was extracted from epididymal and testicular tissues using the RNeasy Kit (QIAGEN, Hilden, Germany) following the manufacturer’s instructions. Genomic DNA contamination was removed using the DNase Kit (TIANGEN, Beijing, China). RNA concentration and purity were assessed using a NanoDrop 2000 spectrophotometer (Thermo Fisher Scientific, Waltham, MA, USA), and RNA integrity was evaluated using a 2100 Bioanalyzer (Agilent, Santa Clara, CA, USA). RNA-seq libraries were constructed as described previously [27]. Library concentration and insert size were measured using a Qubit 2.0 fluorometer (Invitrogen, Carlsbad, CA, USA) and an Agilent 2100 Bioanalyzer (Agilent Technologies, Santa Clara, CA, USA), respectively, and the effective concentration was quantified by quantitative PCR (qPCR). The qualified libraries were sequenced on an Illumina NovaSeq 6000 platform, generating 150 bp paired-end reads.

Raw reads were trimmed using Trimmomatic v0.39 [29] and assessed for quality using FastQC v0.11.9 [30]. Clean reads were mapped to the ovine reference genome (ARS-UI_Ramb_v3.0) [5] using HISAT2 v2.2.1 with default parameters [31] (Table S2). Gene-level read counts were quantified using featureCounts v2.0.1 [32], and expression levels were normalized to transcripts per million (TPM).

### 2.4. Sample Clustering and Quality Control

Principal component analysis (PCA) was performed on TPM-normalized expression data using the prcomp function. The top two principal components were visualized to assess sample clustering patterns. Hierarchical clustering was performed using Euclidean distance and Ward’s linkage method, and heatmaps were generated using the pheatmap package. All analyses were conducted in R version 4.2.0.

### 2.5. Differential Expression Analysis of Y Chromosome Genes

Differential expression analysis was performed using DESeq2 (v1.36.0) [33]. For each pairwise comparison, genes with zero counts across all samples within that comparison were filtered out prior to analysis to remove transcripts with no detectable expression. Four pairwise comparisons were conducted at 6 months of age: LT vs. ST, LE vs. SE, and CT vs. NT. An additional comparison was performed between sexually mature (SM, 6–12 months) and prepubertal (PP, 0–3 months) samples using the 11 developmental time series samples. For each pairwise comparison, all samples were processed within the same batch, minimizing potential technical variation within each comparison. Differentially expressed genes were defined as those with |log_2_(fold change)| > 1 and Benjamini–Hochberg false discovery rate (FDR) adjusted *p*-value < 0.05.

### 2.6. Temporal Expression Pattern Analysis of Y-Chromosomal Genes

Only Y-linked genes located within the MSY were retained for analysis, with tRNA-annotated sequences excluded. Clustering analysis was performed using the Mfuzz package (v2.66.0) in R [34]. The raw TPM expression matrix was log_2_-transformed with a pseudocount of 1 (log_2_(TPM + 1)) and standardized (mean = 0, sd = 1) prior to analysis. The number of clusters was set to six based on the Dmin value, and the minimum membership threshold was set to 0.5.

### 2.7. X–Y Gametolog Expression Analysis

Based on annotation quality and reported reproductive functions, twelve gametolog pairs were selected for further analysis (*AMELY/AMELX*, *DDX3Y/DDX3X*, *EIF1AY/EIF1AX*, *EIF2S3Y/EIF2S3X*, *UTY/KDM6A*, *OFD1Y/OFD1X*, *SRY/SOX3*, *SHROOM2Y/SHROOM2X*, *RBMY/RBMX*, *USP9Y/USP9X*, *ZRSR2Y/ZRSR2X*, and *ZFY/ZFX*).

For each 6-month-old sample (testis: NT, LT, ST, CT; epididymis: LE, SE), TPM expression values of X- and Y-linked genes were extracted. To avoid undefined logarithms for zero expression values, a pseudocount of 0.1 was added to all TPM values. For each gametolog pair, scatter plots were generated on log10-transformed axes, and Spearman’s rank correlation coefficient (ρ) was calculated to quantify the correlation between X and Y expression levels.

### 2.8. Statistical Analysis

Statistical analyses were performed using SPSS 26.0 software (IBM, Armonk, NY, USA) and Python (v3.10.13) with the SciPy and statsmodels packages, with a significance level of α = 0.05. Quantitative data were presented as mean ± standard deviation (SD). For comparisons, one-way analysis of variance (ANOVA) was used, followed by Tukey’s Honestly Significant Difference (HSD) test for post hoc pairwise multiple comparisons.

For correlation analysis, Spearman’s rank correlation coefficients were calculated for X–Y homologous gene pairs within testis and epididymis tissues separately. Gene pairs with no expression were excluded from the analysis for that tissue. To compare correlation strengths between testis and epididymis, Fisher’s z-test followed by a two-sample z-test was applied. The FDR procedure was used to correct for multiple testing within each tissue and across tissue comparisons, with q < 0.05 considered statistically significant.

## 3. Results

### 3.1. Phenotypic Characterization and Y-Linked Transcriptional Clustering

**Figure 1:**
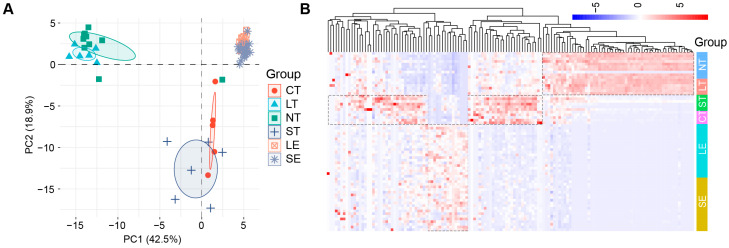
Principal component analysis (PCA) and hierarchical clustering heatmap of transcriptome profiles across different phenotypic groups of Hu sheep. (**A**) PCA plot illustrating the overall transcriptomic similarity among samples from different groups. The first two principal components (PC1 and PC2) explain 42.5% and 18.9% of the total variance, respectively. Each point represents an individual biological replicate, with colors and shapes indicating group membership. The ellipses denote the 95% confidence interval for each group’s distribution. (**B**) Heatmap of normalized gene expression across all samples. Rows represent genes, and columns represent individual samples labeled by group. The color scale indicates normalized expression levels (blue = low, red = high). Hierarchical clustering trees for genes and samples are shown, reflecting the similarity in expression patterns.

Testicular and epididymal weights varied significantly among phenotypic groups (6-month-old Hu sheep: LT, ST, CT, NT, LE, and SE; *p* < 0.05), with minor intergroup variation in body weight (Table 1). Histological examination confirmed that ST testes exhibited severe spermatogenic impairment compared with LT (Figure S1), supporting the validity of our weight-based grouping. Across all specimens, overall grouping was governed by tissue type and animal age, with no evident segregation driven by sequencing batch. Hierarchical clustering based on Y-linked gene expression clearly separated LT and NT testes from ST and CT testes, while LE and SE epididymides showed highly similar transcriptional profiles (Figure 1A,B). When postnatal developmental samples were included, LT and NT testes clustered alongside SM testes. Notably, ST testes did not cluster with either PP or SM testes and displayed higher transcriptional similarity to CT testes (Figure S2).

### 3.2. Developmental Expression Trajectories of Sheep Y-Linked Genes in Testes

**Figure 2:**
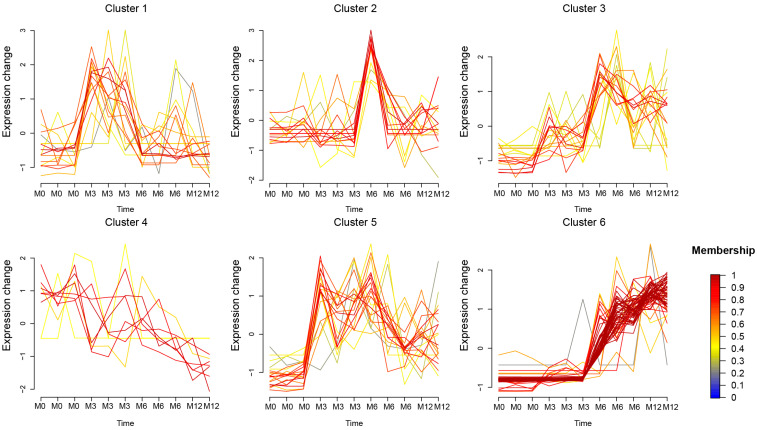
Temporal expression profile clustering of genes across developmental stages in Hu sheep. Gene expression profiles were clustered into six distinct patterns (Cluster 1–6) based on their normalized expression changes across developmental time points (M0, birth (*n* = 3); M3, 3 months (*n* = 3); M6, 6 months (*n* = 3); M12, 12 months (*n* = 2)). Each line represents an individual gene, with color intensity indicating its membership score in the corresponding cluster (ranging from 0, low membership, to 1, high membership, as shown in the color bar on the right). The *y*-axis represents the normalized expression change, and the *x*-axis represents the developmental time points.

To characterize developmental expression dynamics of Y-linked genes, we analyzed testicular transcriptomes from four postnatal stages (0, 3, 6, and 12 months). Clustering analysis assigned 134 expressed Y-linked genes to six co-expression modules with distinct temporal trajectories (Figure 2). The six clusters contained 16, 16, 18, 9, 20, and 55 genes, respectively, with Cluster 6 representing the largest module (Figure 2). Cluster 6 was dominated by multicopy Y-linked gene families, including *ZNF280BY* (25 copies), *HSFY* (12 copies), *TSPY3* (5 copies), and *PRAMEY* (1 copy). Based on temporal expression profiles, Y-linked genes could be broadly classified into three developmental patterns: progressively increased expression (Clusters 3 and 6), progressively decreased expression (Cluster 4), and stage-specific expression (Clusters 1, 2, and 5).

### 3.3. Shared and Phenotype-Specific Y-Linked Transcriptional Signatures

**Figure 3:**
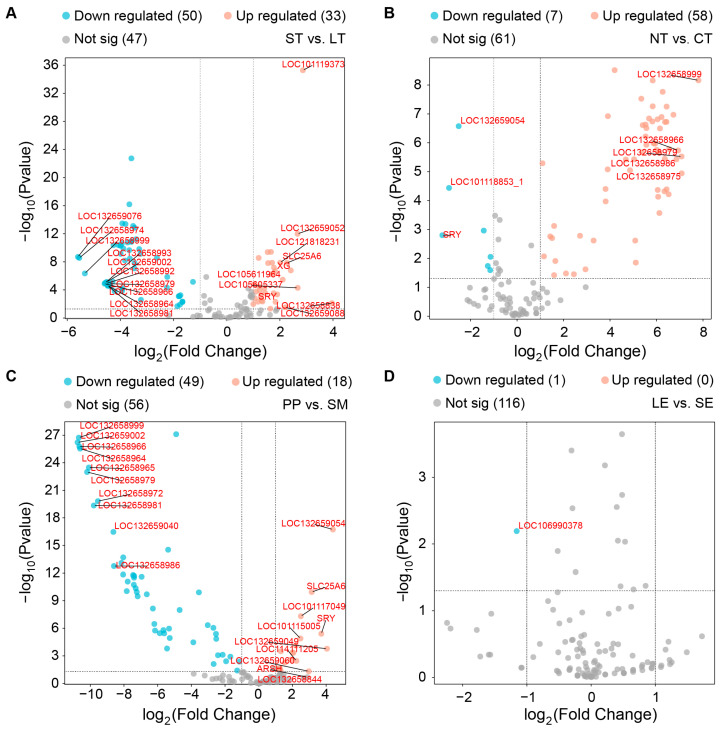
Volcano plots showing differentially expressed genes (DEGs) in four pairwise comparisons. (**A**) ST vs. LT (small testis vs. large testis); (**B**) NT vs. CT (normal testis vs. cryptorchidism); (**C**) PP vs. SM (post-pubertal vs. sexually mature testis, or specify your exact group definitions); (**D**) LE vs. SE (large epididymis vs. small epididymis).

Having established the developmental expression landscape of Y-linked genes, we next examined their association with testicular phenotypic variation. Differential expression analysis identified 83, 65, 1, and 67 Y-linked DEGs between LT vs. ST, NT vs. CT, LE vs. SE, and SM vs. PP groups, respectively (Figure 3; Tables S3–S5). The largest transcriptional differences were observed in testicular comparisons, whereas only a single DEG was detected between epididymal groups.

Among these, 41 DEGs were shared across ST vs. LT, NT vs. CT, and SM vs. PP comparisons, of which 36 were multicopy Y-linked genes including *HSFY*, *ZNF280BY*, and *TSPY3* (Figure S3). Clustering analysis grouped LT, NT, and SM samples together, whereas ST, CT, and PP samples formed a separate cluster (Figure S2). Furthermore, five unique DEGs (*ANOS1*, *ZRSR2Y*, *OFD1Y*, *PPP2R3B*, *LOC121818143*) were identified in the testis versus cryptorchid comparison, and 12 unique DEGs (including *ZFY* and *EIF2S3Y*) were identified in the SM vs. PP comparison (Figure S2). A summary of the key Y-linked DEGs across all contrasts is provided in Table S6.

### 3.4. Co-Expression Patterns of X–Y Gametolog Pairs Across Testicular States

**Figure 4:**
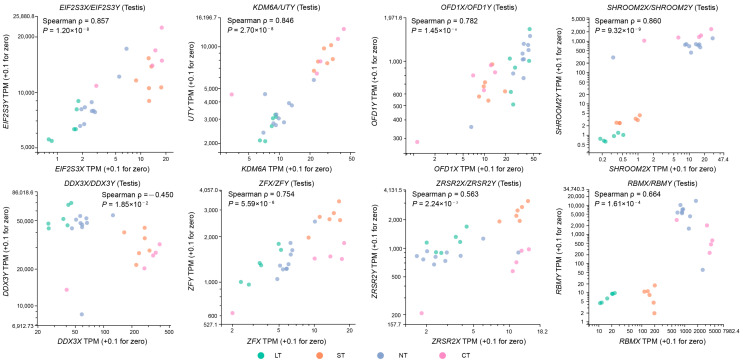
Correlation analysis of gene expression between X- and Y-chromosomal homologs in Hu sheep testis tissue. Scatter plots showing the Spearman correlation between the expression levels (TPM, transcripts per million) of X-chromosomal genes and their corresponding Y-chromosomal homologs in testis samples. TPM values were adjusted by adding 0.1 to handle zero-expression values before plotting. Each point represents an individual sample, color-coded by group: LT (large testis, green), ST (small testis, orange), NT (normal testis, blue), and CT (cryptorchidism, pink). The Spearman correlation coefficient (ρ) and corresponding *p*-value are provided for each pair.

To assess whether the expression relationship between X-linked and Y-linked gametolog pairs varies across testicular phenotypes, Spearman’s rank correlation coefficients were calculated for 12 core X–Y pairs in the LT, ST, NT, and CT groups (Figure 4). In the testis, 8 out of 12 pairs (66.7%) showed significant expression correlations (*p* < 0.05), including seven positively correlated pairs and one negatively correlated pair (*DDX3X/DDX3Y*). The seven positively correlated gene pairs showed Spearman’s rank correlation coefficients ranging from 0.56 to 0.86 (Figure 4). Specifically, three pairs (*SHROOM2X/SHROOM2Y*, *EIF2S3X/EIF2S3Y*, and *KDM6A/UTY*) showed the strongest correlations (ρ > 0.84, *p* < 1.00 × 10^−7^), with group-specific expression patterns (low in LT, moderate in ST, high in NT and CT). Two pairs (*OFD1X/OFD1Y* and *ZFX/ZFY*) showed strong correlations (0.75 < ρ < 0.78, *p* < 1.00 × 10^−5^), with significantly higher expression in CT than in other groups (*p* < 0.05). Two pairs (*RBMX/RBMY* and *ZRSR2X/ZRSR2Y*) showed moderate correlations (0.56 < ρ < 0.66, *p* < 0.01), with higher expression in NT than in other groups (Figure S4).

Notably, *DDX3X/DDX3Y* displayed a significant negative Spearman’s rank correlation (ρ = −0.450, *p* = 0.0185), in contrast to the positive correlations observed for the other seven pairs. Across all groups, *DDX3Y* expression was consistently higher than *DDX3X*. The Y/X ratio showed a progressive decline from LT through NT and ST to CT (one-way ANOVA, *p* < 0.05; Table S7). *DDX3Y* expression decreased progressively from LT to CT, while *DDX3X* expression remained low in LT and NT but was markedly elevated in ST and CT (Table S7).

In the epididymis, four pairs showed significant positive correlations (ρ > 0.3, *p* < 0.05, FDR q < 0.05) (Figure S5; Table S8). Two pairs (*DDX3X/DDX3Y* and *ZRSR2X/ZRSR2Y*) showed strong correlations (ρ > 0.50, *p* < 0.001). Three pairs (*EIF2S3X/EIF2S3Y*, *ZFX/ZFY*, and *SHROOM2X/SHROOM2Y*) showed moderate correlations (0.30 < ρ < 0.50, *p* < 0.05). The remaining seven pairs showed no significant correlation; *EIF1AX/EIF1AY* and *RBMX/RBMY* lacked detectable Y-linked expression in the epididymis (Figure S5). In the epididymis, the *DDX3X/DDX3Y* pair completely reversed its testicular regulatory pattern, exhibiting a significant positive correlation (ρ = 0.607, *p* = 3.29 × 10^−5^). *DDX3Y* expression was significantly higher in SE than in LE (*p* = 0.00274), whereas *DDX3X* showed no intergroup difference (*p* = 0.1346), resulting in a significantly higher Y/X ratio in SE (*p* = 0.00493).

Cross-tissue comparison using Fisher’s z-test further confirmed that correlation strengths for *DDX3X/DDX3Y* (Z = −4.535, FDR q = 5.75 × 10^−5^), along with four other pairs (*KDM6A*/*UTY*, *SHROOM2X*/*SHROOM2Y*, *OFD1X*/*OFD1Y*, and *EIF2S3X*/*EIF2S3Y*), differed significantly between testis and epididymis (FDR q < 0.05; Table S9), indicating male tissue-specific regulatory divergence of X–Y gametologs.

## 4. Discussion

Y-chromosome genes have conventionally been investigated within the frameworks of sex determination and spermatogenesis [3,35]. Testicular size is a potential morphological biomarker associated with spermatogenic function in sheep [36]. In sheep, Y-linked genes are also closely linked to testicular size [37]. For example, *DDX3Y* expression in the testis positively correlates with testis size in Hu sheep, and a novel SNP (g.12657 C>A) in its 3′UTR is significantly associated with testis size [38]. Given their male-specific inheritance and predominant expression in reproductive tissues [39], Y-linked genes may reflect the testicular functional status [4], marking both normal developmental progression and pathological aberrations. Despite these established genetic associations, the transcriptional dynamics of Y-linked genes across diverse testicular functional states, including size variation, developmental progression, and pathological conditions, have remained poorly characterized. To bridge this knowledge gap, we utilized multi-faceted RNA-seq datasets that encompass testicular phenotypes at six months of age (LT, ST, NT, CT, LE, and SE) as well as a continuous developmental series from 0 to 12 months.

### 4.1. Y-Linked Transcriptional Profiles Reflect Testicular Developmental Status

In six-month-old Hu sheep, NT and LT groups exhibited similar Y-linked gene expression patterns, whereas ST clustered together with CT groups. Previous studies have primarily focused on differences between cryptorchid and normal testes [27], leaving the relationship between cryptorchidism and intrascrotal small testes largely unexplored. The prevailing hypothesis has been that smaller testes resemble immature testes, representing developmental delay rather than dysfunction [40]. In our study, sexually mature small testes clustered consistently with cryptorchid testes rather than with immature testes. This clustering pattern is consistent with the possibility that reduced testicular size in sexually mature rams reflects a developmental abnormality rather than simple developmental delay. This interpretation is consistent with a previous autosomal gene expression study [41]. Histopathologically, cryptorchid testes are characterized by reduced seminiferous tubule diameter, germ cell loss, Leydig cell hypoplasia, and interstitial fibrosis [27,42]. Similar degenerative features, including arrested spermatogenesis and Sertoli cell abnormalities [43], have been documented in both cryptorchid and hypoplastic testes across various species [44,45]. Collectively, these findings support the concept that Y-linked transcriptional signatures may serve as informative molecular indicators of testicular developmental status in sheep.

### 4.2. Multicopy Y-Linked Genes Represent a Core Transcriptional Component of Testicular Development

Multicopy and single-copy Y-linked genes exhibited markedly different transcriptional behaviors across developmental and pathological testicular conditions [46]. Most DEGs shared among prepubertal testes, small-testis, and cryptorchid testes belonged to multicopy MSY gene families, including *ZNF280BY*, *PRAMEY*, *HSFY*, and *TSPY3*. The recurrent involvement of these genes across biologically distinct contexts suggests that multicopy Y-linked families may be particularly responsive to changes in testicular functional status. This pattern is consistent with the evolutionary expansion of ampliconic genes on the mammalian Y chromosome [5,47], which is thought to compensate for the constraints imposed by long-term suppression of recombination. In contrast, single-copy genes showed condition-specific expression patterns: *ANOS1* uniquely in NT vs. CT, as well as *ZFY* and *EIF2S3Y* uniquely in SM vs. PP. These patterns align with their known functions: *ANOS1* mutations cause Kallmann syndrome with cryptorchidism [48], and *ZFY* and *EIF2S3Y* knockouts impair mouse spermatogenesis [4,49]. Together, these findings suggest that multicopy and single-copy Y-linked genes may play distinct roles in testicular physiology.

### 4.3. Tissue-Specific Expression Relationships of X–Y Gametolog Pairs

Most X–Y gametolog pairs showed positive expression correlations in the sheep testes, suggesting that coordinated transcription between homologous X- and Y-linked genes is maintained across different testicular states. Similar expression patterns have been reported in other mammals and are generally considered indicative of conserved regulatory relationships between X- and Y-linked counterparts [12,50]. Notably, the relative expression levels of several gametolog pairs varied among testicular phenotypic groups, suggesting that sex-chromosome transcriptional balance changes with testicular developmental status.

In contrast to most gametolog pairs, *DDX3X/DDX3Y* displayed opposite correlation patterns in testes and epididymides, exhibiting a negative correlation in testis but a positive correlation in epididymal tissue. Previous studies have shown that *DDX3Y* plays important roles in spermatogenesis and male germ-cell development [51], whereas *DDX3X* performs broader cellular functions [52]. The tissue-specific expression relationship observed here may therefore reflect differences in cellular composition and RNA metabolism [53]. Furthermore, the negative correlation between *DDX3X* and *DDX3Y* became progressively weaker from large and normal testes to small and cryptorchid testes, indicating that the transcriptional relationship between these genes changes across testicular states. Although the biological basis of this pattern remains unclear, the findings suggest that *DDX3X/DDX3Y* regulation is associated with testicular developmental status and warrants further investigation.

### 4.4. Study Limitations and Future Perspectives

Several limitations should be acknowledged. First, the present study is based primarily on transcriptomic analyses and therefore cannot establish causal relationships between gene expression changes and testicular dysfunction. Second, some comparison subgroups contain a limited number of biological replicates, highlighting the need for future validation with enlarged sample sizes. Third, single-cell data were unavailable, limiting cellular mechanistic inference. Future application of single-cell RNA sequencing will enable precise dissection of cell populations responsible for spermatogenic defects and allow direct testing of cell-type-specific expression biases of X–Y gametologs (e.g., *DDX3X/DDX3Y*). Finally, validation in larger populations and additional sheep breeds will be necessary to evaluate the generality of the identified Y-linked signatures. Future studies integrating transcriptomic, genomic and cellular data will further clarify the biological functions of key Y-linked genes and their potential application as molecular indicators of male reproductive performance.

## 5. Conclusions

In summary, this study systematically characterized Y-linked gene expression across postnatal testicular development, testicular phenotypic variation, and cryptorchidism in sheep. Y-linked transcriptional profiles clearly distinguished normal and impaired testicular states, with small testes exhibiting expression patterns more similar to cryptorchid testes than to normal developmental stages. Multicopy Y-linked gene families, including *ZNF280BY*, *HSFY*, *PRAMEY*, and *TSPY3*, showed coordinated developmental regulation and represented the major transcriptional component associated with testicular maturation. In addition, most X–Y gametolog pairs displayed positive expression relationships, whereas *DDX3X/DDX3Y* exhibited a distinct tissue-specific correlation pattern between testis and epididymis. These findings provide new insights into Y-chromosome transcriptional regulation in sheep and highlight the potential value of Y-linked transcriptional signatures as molecular correlates of testicular developmental status; further functional validation is required to confirm their utility as biomarkers predictive of male reproductive capacity.

## Figures and Tables

**Table 1:** Testicular and epididymal phenotypic information in 6-month-old rams.

Group	Number	Total Testis Weight (g)	Total Epididymis Weight (g)	Live Weight (kg)
Large testis (LT)	6	439.98 ± 21.28 ^a^	58.53 ± 4.03 ^a^	55.22 ± 1.05 ^a^
Small testis (ST)	6	74.38 ± 6.92 ^e^	27.33 ± 2.82 ^c^	55.38 ± 1.82 ^a^
Normal testis (NT)	10	223.43 ± 114.49 ^c^	35.03 ± 9.28 ^c^	42.95 ± 4.45 ^b^
Cryptorchidism (CT)	5	205.28 ± 73.82 ^c^	26.18 ± 5.52 ^c^	43.20 ± 1.51 ^b^
Large epididymis (LE)	20	350.82 ± 62.71 ^b^	50.73 ± 3.18 ^b^	50.90 ± 6.18 ^a^
Small epididymis (SE)	20	134.83 ± 48.37 ^d^	22.56 ± 2.48 ^d^	46.55 ± 4.59 ^ab^

Data are presented as mean ± standard deviation (SD). The number of biological replicates (n) for each group is listed in the table. Values with different superscript letters in the same column differ significantly (*p* < 0.05).

## Data Availability

The raw transcriptome sequencing data generated in this study have been deposited in the NCBI Sequence Read Archive (SRA) under BioProject accession numbers PRJNA1199078 and PRJNA763055. Additional data are available from the corresponding author upon reasonable request.

## Supplementary Materials

The following supporting information can be downloaded at: https://www.mdpi.com/article/10.3390/ani16132107/s1, Figure S1: Haematoxylin and eosin (HE) staining of testicular cross-sections from 6-month-old Hu sheep; Figure S2: Y-linked gene expression patterns and global transcriptomic differences in phenotypically distinct testes and epididymides across developmental stages of Hu sheep; Figure S3: Venn diagram showing the overlap of differentially expressed Y-linked genes across three pairwise comparisons; Figure S4: Correlation analysis of gene expression between X- and Y-chromosomal homologs in Hu sheep testis tissue; Figure S5: Correlation analysis of gene expression between X- and Y-chromosomal homologs in Hu sheep epididymis tissue; Table S1: Sample metadata for transcriptomic analysis; Table S2: Summary of RNA-seq read mapping statistics for all samples; Table S3: Y-linked differentially expressed genes between large and small testes groups; Table S4: Y-linked differentially expressed genes between cryptorchid and normal testes; Table S5: Y-linked differentially expressed genes between sexually mature and immature groups; Table S6: Key Y-linked differentially expressed genes (Y-DEGs) across experimental contrasts and their annotated functions; Table S7: Descriptive statistics and intergroup comparisons of *DDX3Y*, *DDX3X* expression levels and Y/X ratio in four testis groups; Table S8: Spearman correlation coefficients between X–Y homologous gene pairs in testis and epididymis tissues; Table S9: Cross-tissue comparison of Spearman correlation coefficients between testis and epididymis tissues.

## Abbreviations

The following abbreviations are used in this manuscript:

CT	Cryptorchidism
DEG	Differentially expressed gene
LE	Large epididymis
LT	Large testis
MSY	Male-specific region of the Y chromosome
NT	Normal testis
PCA	Principal component analysis
PP	Prepubertal
SE	Small epididymis
SM	Sexually mature
ST	Small testis
TPM	Transcripts per million
