# Supplementary material for: Y-Linked Expression Signatures Distinguish Dysfunctional Testicular States in Sheep
Source: Animals (Basel). 2026 Jul 7;16(13):2107. doi: 10.3390/ani16132107 (PMC13360604; doi:10.3390/ani16132107)
Supplement: Supplementary file 1 [file animals-16-02107-s001.zip › Supplementary Figures.pdf]

Supplementary Figures

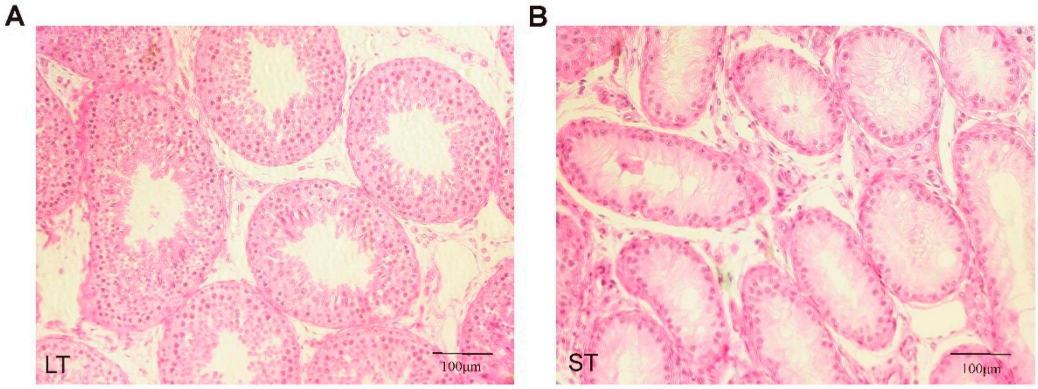

Figure S1. Haematoxylin and eosin (HE) staining of testicular cross-sections from 6-month-old Hu sheep. (A) Large testis (LT) group: Robust, intact seminiferous tubules with complete multi-layered spermatogenic cells and abundant mature sperm inside the tubular lumen. (B) Small testis (ST) group: Atrophic, collapsed seminiferous tubules, drastically reduced spermatogenic cell layers, massive depletion of germ cells, and absence of mature sperm in lumens. Scale bar = 100 μm.

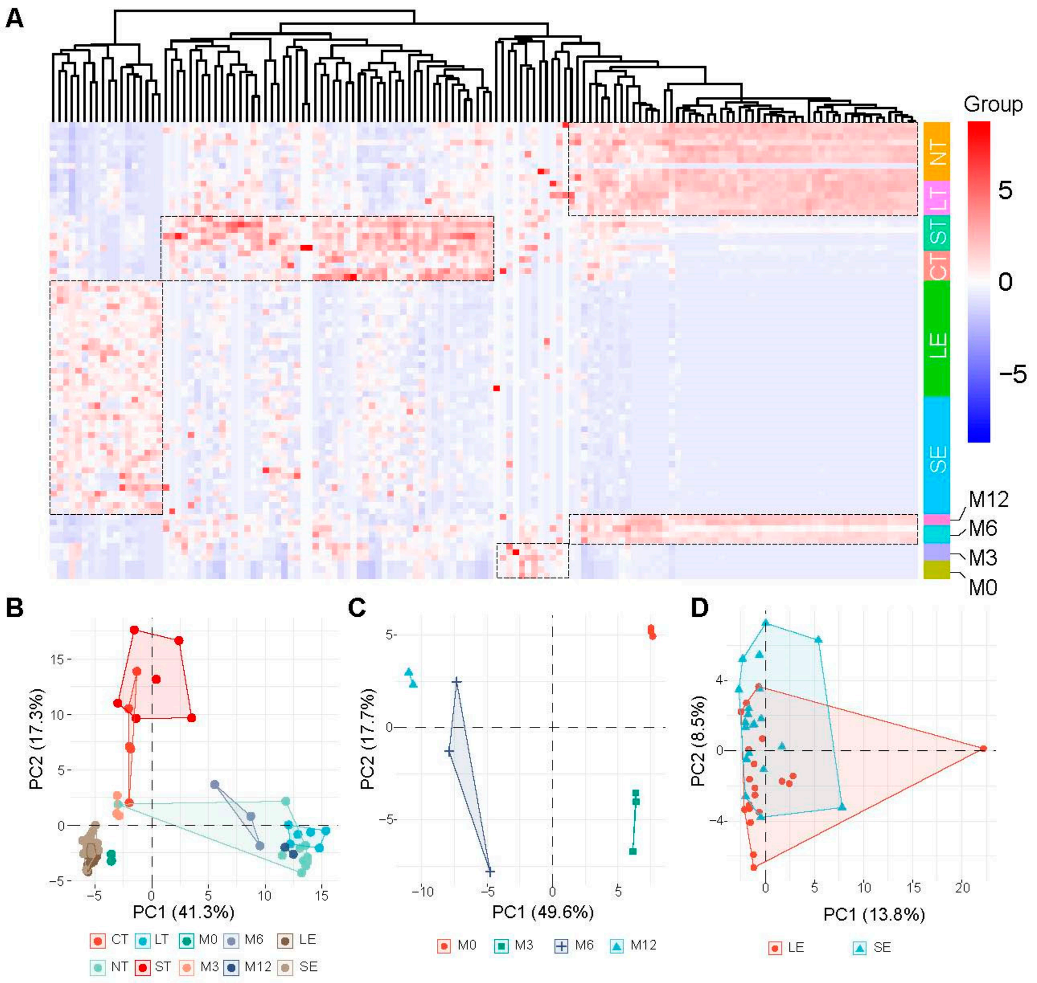

Figure S2. Y-linked gene expression patterns and global transcriptomic differences in phenotypically distinct testes and epididymides across developmental stages of Hu sheep. (A) Hierarchical clustering heatmap of Y-linked gene expression profiles across developmental stages and phenotypic groups in Hu sheep. Heatmap showing the normalized expression patterns of Y-linked genes across samples from different phenotypic groups (NT, LT, ST, CT, LE, SE) and developmental time points (M0, birth; M3, 3 months; M6, 6 months; M12, 12 months). (B) Principal component analysis (PCA) of all samples based on the full transcriptome dataset. (C) PCA plot stratified only by developmental stages (M0, M3, M6, M12). (D) PCA plot restricted to epididymal samples (LE vs. SE).

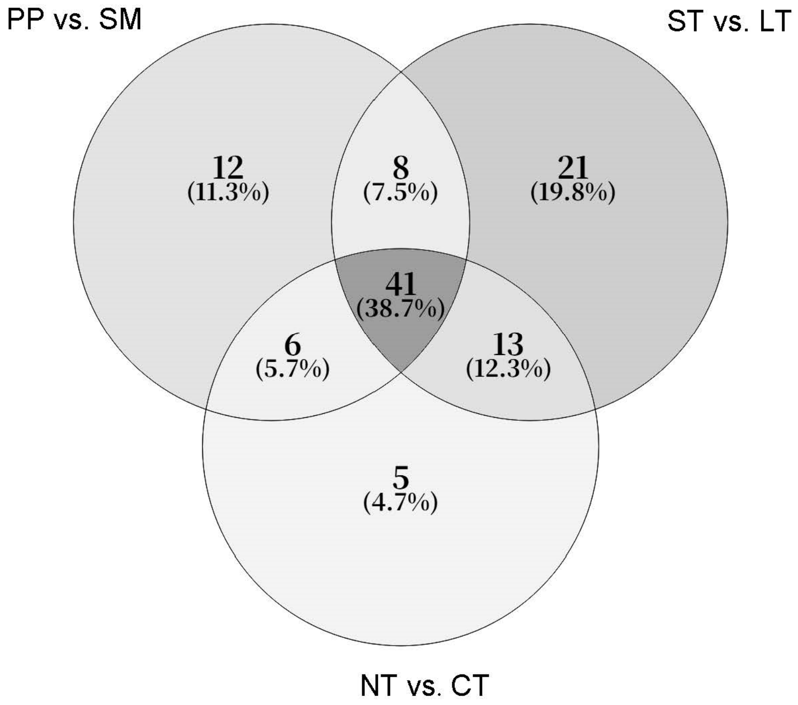

Figure S3. Venn diagram showing the overlap of differentially expressed Y-linked genes across three pairwise comparisons. Venn diagram illustrating the number of unique and shared differentially expressed Y-linked genes (DEGs) identified in three pairwise comparisons: PP vs. SM (pre-pubertal vs. sexually mature testis), ST vs. LT (small vs. large testis), and NT vs. CT (normal vs. cryptorchid testis).

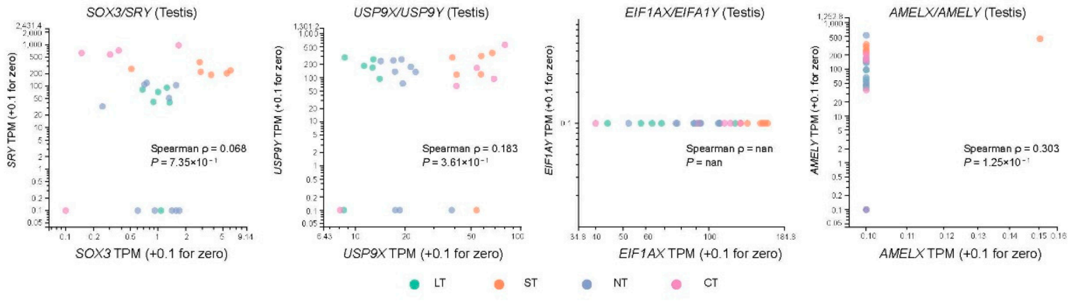

Figure S4. Correlation analysis of gene expression between X- and Y-chromosomal homologs in Hu sheep testis tissue. Scatter plots illustrating the Spearman correlation between the expression levels (TPM, transcripts per million) of four pairs of X- and Y-chromosomal homologs in testis samples: *SOX3/SRY*, *USP9X/USP9Y*, *EIF1AX/EIF1AY*, and *AMELX/AMELY*. TPM values were adjusted by adding 0.1 to

handle zero-expression values before plotting. Each point represents an individual sample, color-coded by group: LT (large testis, green), ST (small testis, orange), NT (normal testis, blue), and CT (cryptorchidism, pink). The Spearman correlation coefficient (ρ) and corresponding *P*-value are provided for each pair; nan indicates insufficient variation to compute the correlation.

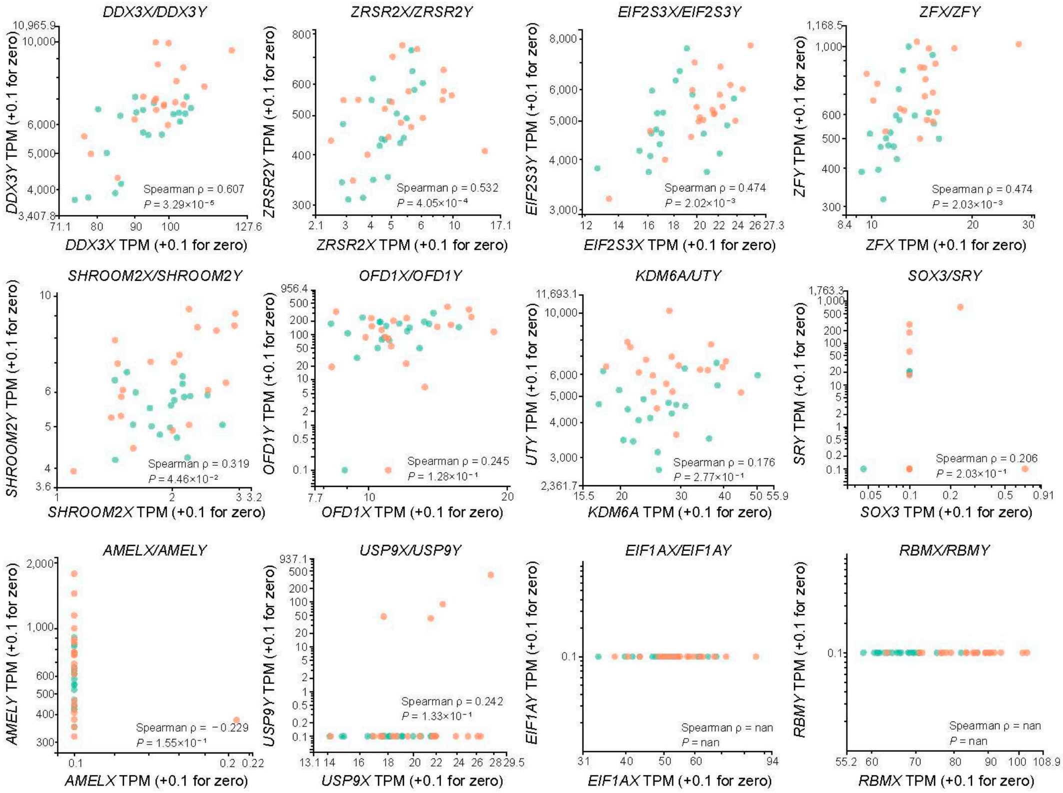

Figure S5. Correlation analysis of gene expression between X- and Y-chromosomal homologs in Hu sheep epididymis tissue. TPM values were adjusted by adding 0.1 to handle zero-expression values before plotting. Each point represents an individual sample, color-coded by group (LT, large testis, green; ST, small testis, orange). The Spearman correlation coefficient (ρ) and corresponding *P*-value are provided for each pair; nan indicates insufficient variation to compute the correlation.
